# Borggreve-Van Nes rotationplasty for infected knee arthroplasty – a case report

**DOI:** 10.3109/17453671003667168

**Published:** 2010-04-06

**Authors:** Charles E Dumont, André J Schuster, Marie Freslier-Bossa

**Affiliations:** ^1^Orthopedics Clinic, Spital Ziegler, Spitalnetzbern, Bern; ^2^Orthopedics Center, Zürich; ^3^Laboratory for Movement Analysis, Children's University Hospital, BaselSwitzerland

## Introduction

**Figure 1.**
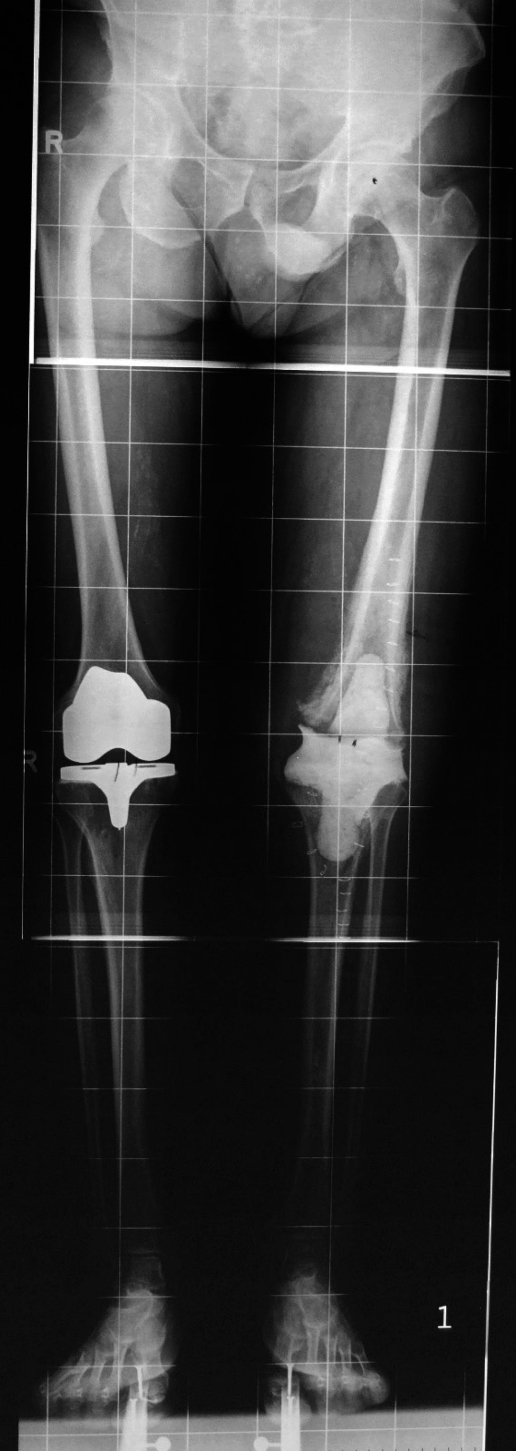
The cement spacer after revision for an infection recurrence following a 2-stage reimplantation for a late infection of the left TKA. The pelvic obliquity is due to the 10-cm leg length discrepancy. There is no loosening of the right TKA.

A 62-year-old patient presented with a recurrence of infection of the left knee following a two-stage TKA reimplantation for the treatment of a late infection of a TKA implanted in 1995. He had had a right knee arthroplasty in 2004, with good function. The new infection was treated by a debridement, the revision prosthesis was removed, and a cement spacer was placed in the 10-cm bone defect (Figure 1). The tibia tubercle and part of the cement spacer were exposed by an anterior 15-cm2 soft tissue defect. Cultures from the wound taken during the procedure showed a monomicrobial infection with methicillin-resistant coagulase-negative staphylococcus. The patient was treated with intravenous vancomycin. A second look 1 week later showed further deep soft tissue necrosis, probably due to cement heat during the polymerization phase of the spacer, as well as further bone necrosis in the distal femur, the proximal tibia, and the osteotomized tibia tubercle. Therapeutic options, which were discussed with the patient, included arthrodesis, resection arthroplasty (joint resection without joint replacement), amputation, or a rotationplasty (Christie et al. 2003). The patient elected to have a rotationplasty after having seen pictures of a patient who had had a rotationplasty and after having been informed about the expected functional outcome (Fuchs et al. 2003).

**Figure 2.**
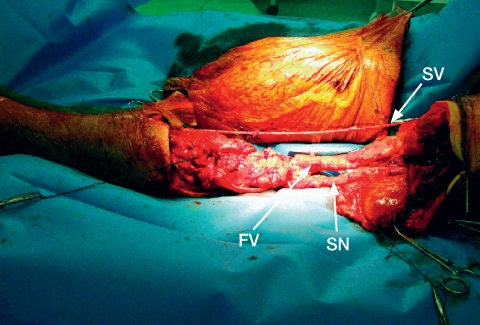
Intraoperative view of the en bloc resection of the knee joint, distal tibia, and proximal femur. Osteotomies of both the femur and the tibia were performed with 2 cm margins above and below the cement spacer endomedullar stumps. The dissected saphenous vein (SV), sciatic nerve (SN), and femoral vessels (FV) are indicated with arrows.

**Figure 3.**
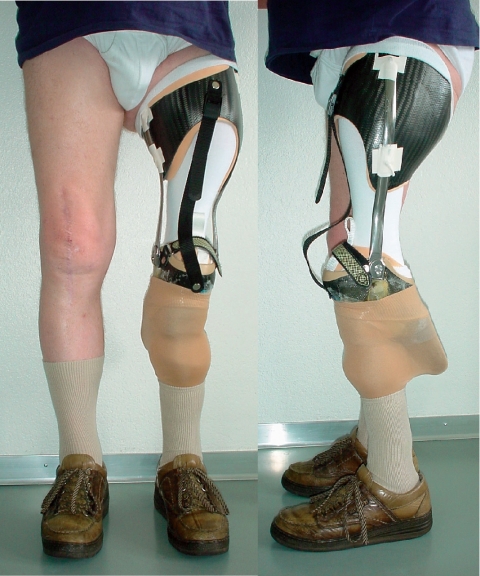
Patient standing with the definitive prosthesis.

**Figure 4.**
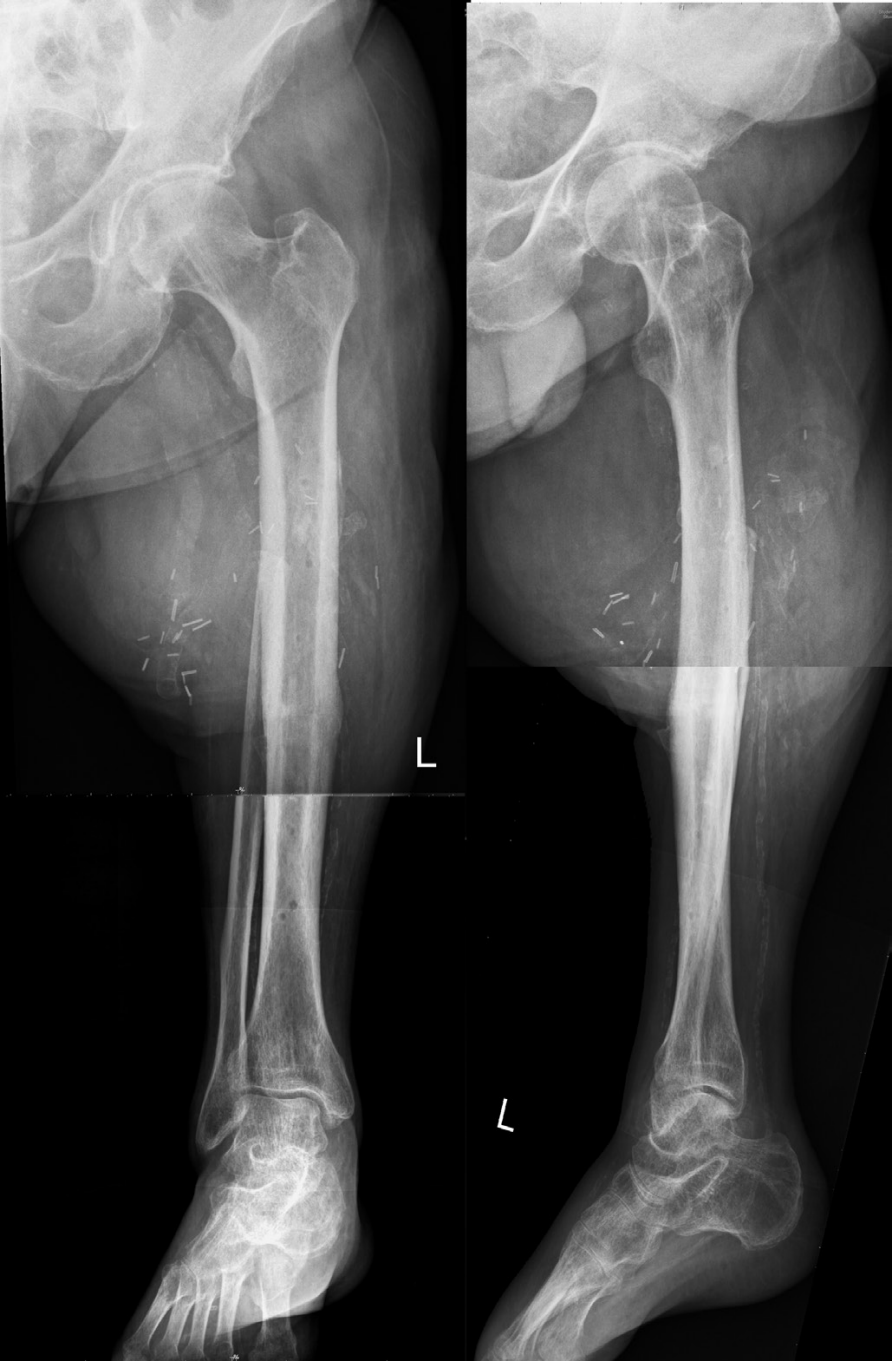
Femoro-tibial fusion at last follow-up.

A rotationplasty was performed according to the technique described by Fuchs and Sim (2004), with the knee “joint” and the distal femur and proximal tibia being resected en bloc (Figure 2). Drainage of a postoperative hematoma was performed on the second postoperative day. The rest of the healing and recovery were uneventful. Time to soft tissue healing was 4 weeks and time to bone consolidation was 4 months. The patient began with full weight bearing with a prosthesis at 6 months. The hardware was removed 1 year after surgery because of pain at the distal edge of the plate.At the last follow-up, 26 months after the rotationplasty, the patient was satisfied with the function of the limb and said that he and his family had no more trouble with the cosmetic appearance of the limb (Figure 3). Radiographs showed healing of the femoro-tibial fusions (Figure 4). The operated limb was pain-free but the contralateral knee was painful after 10 min of walking. He could walk 800 meters without crutches or aid, and could ascend and descend stairs using the handrail. He could drive his car and had no difficulty in getting into and out the car. The active range of the prosthetic knee was 10° to 80°. Dorsiflexion and plantarflexion strength were M5. The free walking speed was 0.75 m/s (averaged on 13 gait cycles). Gait analysis showed a gait pattern that was similar on both sides to an above-the-knee amputation, with both knees extended at heel-strike and no loading response on the side of the rotationplasty because of the stiffness of the prosthesis, and on the contalateral side because of the limited dorsal flexion of the foot.

## Discussion

The Borggreve-Van Nes rotationplasty was initially used to treat shortened limbs and ankylosis of the knee due to tuberculosis, and for congenital defects of the femur (Borggreve 1930, Van Nes 1950). It consists of en bloc resection of the knee, bone shortening, and rotation of 180° to allow the ankle to function as a knee joint. Today, rotationplasty is mainly used for local disease control in young children with bone sarcoma around the knee (Merkel et al. 1991, Winkelmann 1996). Gait analysis has shown preserved ankle proprioception, resulting in a functional outcome of rotationplasty that is better than following TKA (Fuchs et al. 2003). More recently, its usefulness as a salvage procedure for the treatment of posttraumatic osteomyelitis of the distal femur (Krettek et al. 1997) or in infection of massive endoprosthesis in tumor patients (Wicart et al. 2002) has been reported. Salvage procedures for reinfection of revision TKA focus primarily in curing the infection by means of arthrodesis, resection arthroplasty, or above-the-knee amputation. The functional outcome in patients treated with above-the-knee amputation and resection arthroplasty is poor (for review, see Christie et al. 2003). The range of success after arthrodesis differs widely—from 50% to almost 100%, depending on the type of osteosynthesis material used and the extent of bone defect (Hanssen et al. 1995, McQueen et al. 2006).

The function after rotationplasty in our patient was similar to an above-the-knee amputation (Harris et al. 1990), as assessed with gait analysis. The lack of phantom pain because of preservation of the sciatic nerve may indeed argue for inclusion of rotationplasty in the salvage options proposed to non-oncological patients with a history of recurrence of infection and extended bone loss following TKA.
